# Meta-Analysis of Durable Compared to Temporary Left Ventricular Assist Devices Compared to Venoarterial Extracorporeal Membrane Oxygenation for Bridging to Heart Transplantation or Treatment of Primary Graft Dysfunction

**DOI:** 10.31083/RCM45064

**Published:** 2025-12-16

**Authors:** Lars Saemann, Sven Maier, Matthias Kohl, Andreas Simm, Gábor Szabó

**Affiliations:** ^1^Department of Cardiac Surgery, University Hospital Halle, 06120 Halle (Saale), Germany; ^2^Department of Cardiac Surgery, University Hospital Freiburg, Faculty of Medicine, University of Freiburg, 79106 Freiburg, Germany; ^3^Faculty III: Health, Medical Life Sciences, Furtwangen University of Applied Sciences, 78054 Villingen-Schwenningen, Germany; ^4^Department of Cardiac Surgery, University Hospital Heidelberg, 69120 Heidelberg, Germany

**Keywords:** left ventricular assist device, extracorporeal membrane oxygenation, venoarterial, impella, heart transplantation, primary graft dysfunction, bridging to transplant

## Abstract

**Background:**

Patients bridged to heart transplantation (HTx) and patients with primary graft dysfunction (PGD) after HTx are typically treated with circulatory support. However, the survival of patients in both indications might depend on the type of circulatory support. Thus, this meta-analysis aimed to investigate the survival of HTx patients supported during bridging with a durable left ventricular assist device (d-LVAD), a temporary LVAD (t-LVAD), or venoarterial extracorporeal membrane oxygenation (VA-ECMO). We also investigated the survival rate of patients with PGD by type of circulatory support device.

**Methods:**

We performed a random-effects meta-analysis.

**Results:**

We included four studies evaluating bridging to HTx (n = 1678 patients) and three studies for the PGD analysis (n = 35 patients). The 1-year survival after HTx was significantly higher in patients bridged with a t-LVAD (92.7%; 95% confidence interval (CI): 89.2 to 95.6%; *p* = 0.027) and with a d-LVAD (86.8%; 95% CI: 75.8 to 94.8%; *p* = 0.001) compared to VA-ECMO (71.6%; 95% CI: 63.7 to 78.9%). The 30-day survival in patients with PGD and t-LVAD was 100% (95% CI: 59.2–100%), while with PGD and VA-ECMO, survival was 92.4% (95% CI: 66 to 100%).

**Conclusions:**

Both d-LVAD and t-LVAD bridging methods appear to have comparable 1-year survival rates, which are higher than those after VA-ECMO bridging. Nonetheless, more prospective clinical studies are needed to investigate outcomes after using circulatory support devices for PGD after HTx. The PROSPERO registration: CRD420251149065, https://www.crd.york.ac.uk/PROSPERO/view/CRD420251149065.

## 1. Introduction

For patients with end-stage heart failure, heart transplantation (HTx) is the gold standard of therapy. However, patients are increasingly bridged to HTx with a circulatory support system [1]. Most commonly, a durable left ventricular assist device (d-LVAD), which needs to be implanted surgically, has been used for bridging [1]. It unloads the left ventricle through the pump inflow tract implanted through the apex, pumping the blood back into the aorta through the anastomosed outflow tract. Meanwhile, temporary left ventricular assist devices (t-LVAD), which can be inserted percutaneously through the femoral or axillary artery and provide adequate flow rates to maintain circulation in the body, are available in the market. T-LVADs are inserted into the left ventricle through the aorta, and a microaxial pump unloads the left ventricle. These t-LVADs, even when approved only for temporary support, are also increasingly used for bridging the patient to HTx [2]. Finally, veno-arterial extracorporeal membrane oxygenation (VA-ECMO), consisting of an extracorporeal circuit with a pump and an oxygenator, is also used for bridging patients to HTx [1].

Besides bridging to HTx, primary graft dysfunction (PGD) after HTx is another indication of the implantation of a circulatory support system [3, 4]. Currently, a high level of evidence regarding all three circulatory support device options, d-LVAD, t-LVAD, and VA-ECMO, for bridging to HTx and treating PGD does not exist. Thus, this meta-analysis aimed to investigate the survival of patients after HTx supported with d-LVAD, t-LVAD, or VA-ECMO either for bridging to HTx or for treatment of PGD.

## 2. Materials and Methods

### 2.1 Study Design

We performed a meta-analysis according to the Preferred Reporting Items for Systematic Reviews and Meta-analysis statement (PRISMA) [5]. We also used the National Institutes of Health Quality Assessment Tool for Observational Cohort and Cross-Sectional studies and included only studies with a quality rated of “good” (**Supplementary Table 1**) [6]. We included all studies according to the eligibility criteria below, published until September 2023. A registration was performed in PROSPERO (CRD420251149065).

### 2.2 Eligibility Criteria

For the bridging to HTx meta-analysis, we included prospective clinical studies or registry studies with prospectively collected data on adult patients (≥18 years), patients bridged to orthotopic HTx with only left ventricular support, either with a d-LVAD, restricted to the HeartWare, Heartmate II, or Heartmate III, a t-LVAD, restricted to Impella 5.0, Impella 5.5, or CentriMag. For the PGD after HTx meta-analysis, the inclusion criteria corresponded to the criteria mentioned above, with the only difference that instead of patients bridged to transplantation, patients with PGD were included. Multiorgan transplants were not excluded.

In both meta-analyses, we excluded studies that reported a support device use as a bridge to decision, bridge to bridge, bridge to destination, or bridge to another type of support device. We excluded other types of mechanical support devices, such as intra-aortic balloon pumps, older generation LVADs (before HeartMate 2), total artificial hearts, right ventricular support, venovenous-ECMO, and t-LVADs with a smaller circulatory support capacity than 5.0 L/min, because of their incomparability to d-LVADs. We also excluded heterotopic HTx, patients <18 years, and publications that analyzed only specific subpopulations, such as only INTERMACS1 patients. If more than one registry study was based on data from the same registry, we only included the ones with complementary instead of those with overlapping time frames.

### 2.3 Information Sources and Systematic Search

We developed the search strategy with an expert medical science librarian. We searched the electronic databases PubMed via Medline, Social Science Citation Index via Web of Science, Social Science Citation Index Expanded, and the Cochrane Library from the beginning until September 21st, 2023, for the meta-analysis regarding bridging to HTx, and from the beginning until November 16th, 2023, for the meta-analysis regarding PGD. We also applied forward citation tracking and checked the references of relevant articles. We contacted the authors of one publication to clarify the 30-day mortality outcome, but did not receive a response. The full search strategy is presented in the **Supplementary material**.

The publications from each electronic database were exported, including title, abstract, and library/bibliographic information. Then, the database exports were imported to Rayyan, a web app for systematic reviews [7]. Duplicates, suggested by the app, were checked by an author and removed when confirmed.

### 2.4 Study Selection

Inclusion and exclusion criteria and the statement for exclusion reasons were also noted in Rayyan, blinded and thus independently by two reviewers. Dissent was solved by a discussion moderated by a third reviewer in all cases.

### 2.5 Data Collection

Before data collection started, we discussed the variables that needed to be extracted from the articles with two reviewers until an agreement was reached. Data extraction of all included studies was performed independently by two authors. Results were compared, and all varieties were discussed. Another author moderated this process. For more consistency during the review process and minimal risk for errors from a heterogeneous knowledge background, a pilot test was performed with two citations.

### 2.6 Data Items

We collected data based on survival after HTx in the bridged populations and survival after PGD in the PGD population as outcome variables. Further data that was collected was related to recipient characteristics, donor characteristics matching, transplant-related characteristics, and circulatory support device-associated information.

### 2.7 Statistical Analysis

We performed a random-effects meta-analysis using arcsine square root transformed proportions to stabilize the variance [8]. In addition, we performed a meta-regression with group as a moderator for the group comparison. The results are back-transformed to proportions. The meta-analysis was performed using the package metafor [9] of the statistical software R [10].

## 3. Results

**Fig. 1.**
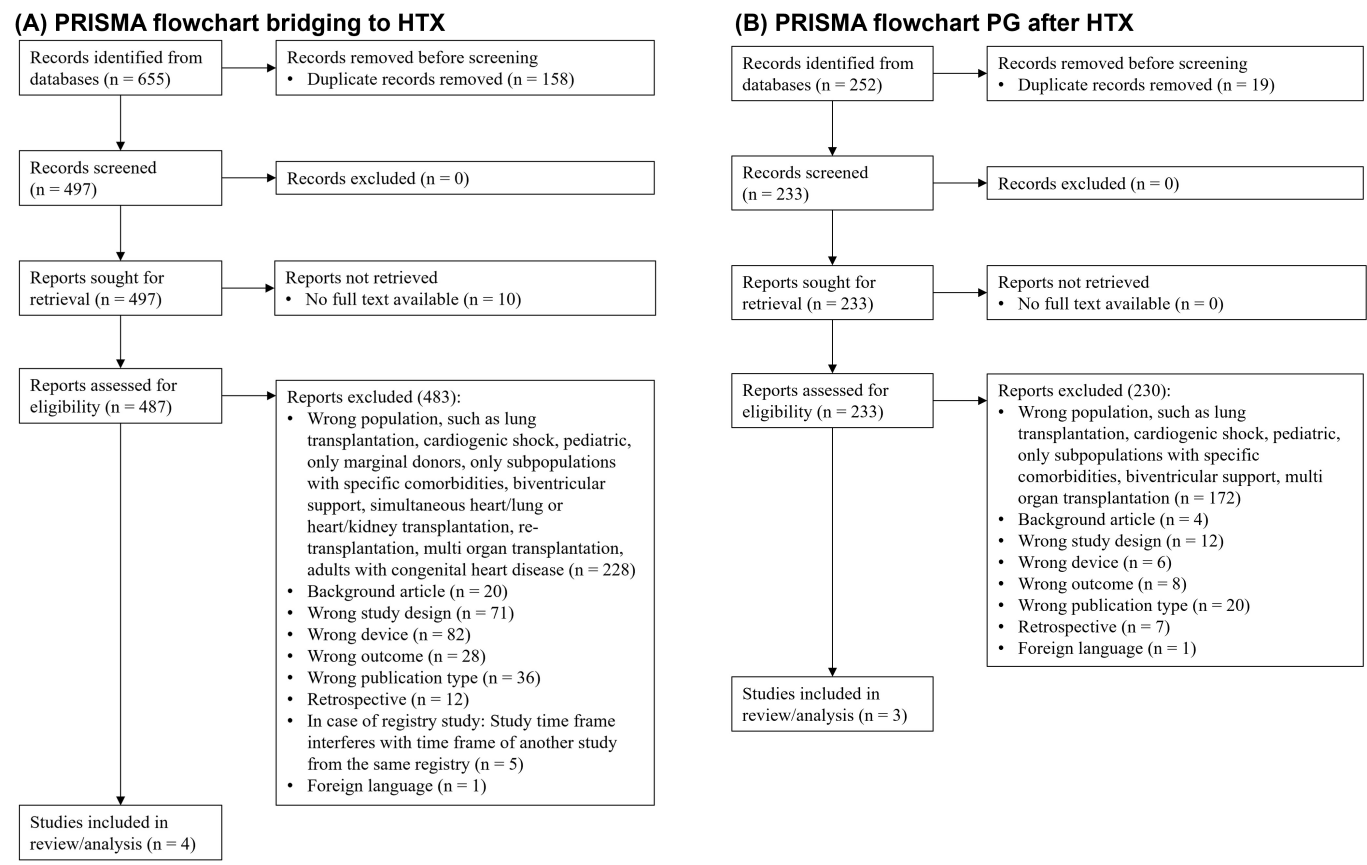
**PRISMA flow chart**. (A) Bridging to HTx. (B) PGD after HTx. HTx, Heart transplantation; PGD, Primary graft dysfunction; PRISMA, Preferred Reporting Items for Systematic Reviews and Meta-analysis statement.

We included four studies for the bridging of HTx analysis and three studies for the PGD after HTx analysis. Reasons for exclusion are shown in the PRISMA flow chart in Fig. 1. The main reasons for exclusion were based on the patient population, device type, study design, and publication type in both analyses. In the selection process of studies for bridging to HTx, we also excluded studies that were based on data from overlapping time frames from the same registry.

For both meta-analyses, we collected data regarding device-, recipient-, donor-, matching-, and transplant-related characteristics (Table 1, Ref. [2, 11, 12, 13] and Table 2, Ref. [14, 15, 16]). However, not every publication contained information on every data item we searched for. However, besides the data items shown in Tables 1,2, we also searched for multiple other data items that were unfortunately not shown in any of the publications. For the bridging to transplantation analysis, this included the recipient characteristics, total bilirubin, cytomegalovirus (CMV) serology pretransplant infection, blood transfusion while on waitlist, and cause of death if death occurred after transplantation. Unstated donor characteristics were donor type (brain death or circulatory), Graft LVEF <50%, total bilirubin, serum creatinine, graft maintenance method (static cold storage and normothermic blood perfusion), graft out-of-body time, and graft blood perfusion time in case of NRP. For the PGD after transplantation analysis, additional unstated items were race, blood type, diabetes mellitus, pretransplant mechanical ventilation, and pretransplant infection regarding recipient characteristics. Regarding donor characteristics, additional missing data items were race, diabetes mellitus, CMV serology, blood type, graft cold ischemic time, and graft maintenance method. Furthermore, the following matching-related characteristics were not given in studies regarding PGD: sex-, race-, HLA-, AB0-, and CMV-matched.

**Table 1.** **Bridging to heart transplantation**.

Study characteristics		Hill *et al*. [2]	Hill *et al*. [2]	Kilcoyne *et al*. [11]	Yin *et al*. [12]	Bedanova *et al*. [13]
Registry study		Yes	Yes	Yes	Yes	No
Registry name		UNOS	UNOS	UNOS	ISHLT	N/A
Time frame of registry study		January 2010–September 2021	January 2010–September 2021	January 2015–June 2021	January 2005–June 2016	N/A
Device-related characteristics						
Device type		t-LVAD	t-LVAD	d-LVAD	VA-ECMO	d-LVAD
Specific designation		Impella 5.0	Impella 5.5	HM3		HM2, HW
Number of patients		251	143	1102	134	48
Post-transplant outcome and complications						
PGD requiring circulatory support					13 (36.1%)	
Post-transplant hospital length of stay, d						35
Recipient characteristics						
Recipient age, years		58	56		44.9	53
Sex, male		203 (80.9%)	126 (86.9%)			94 (45%)
Race						
	White	153 (61%)	91 (62.8%)			
	Black	49 (19.5%)	28 (19.3%)			
	Hispanic	33 (13.1%)	15 (10.3%)			
	Other	16 (6.4%)	11 (7.6%)			
Body mass index, kg/m^2^		26.97	27.33		25.5	27.7
Blood type						
	A				62 (46.3%)	
	AB				10 (7.5%)	
	B				12 (9%)	
	O				50 (37.3%)	
Heart failure etiology						
	Non-Ischemic	163 (64.9%)	103 (71%)		62 (46.6%)	
	Ischemic	74 (29.5%)	36 (24.8%)		44 (33.1%)	
	Congenital	1 (0.4%)	2 (1.4%)		10 (7.5%)	
	Restrictive/Hypertrophic	7 (2.8%)	3 (2.1%)		9 (6.7%)	
	Valvular	0 (0%)	1 (0.7%)		6 (4.5%)	
	Other/missing/unknown	6 (2.4%)	0 (0%)		3 (2.2%)	48 (100%)
Diabetes mellitus		78 (31.1%)	49 (33.8%)		15 (11.3%)	35 (17%)
Creatinine at transplantation, mg/dL		1.13	1.1		1.2	
Pretransplant mechanical ventilation		5 (2%)	1 (0.7%)			
Intravenous inotropes at registration		136 (64.2%)	84 (63.6%)			
Donor characteristics						
Donor age, years		31	32		35.3	42
Sex, male		181 (72.1%)	126 (86.9%)		85 (63.4%)	83 (40%)
Race						
	White	127 (50.6%)	97 (66.9%)			
	Black	38 (15.1%)	20 (13.8%)			
	Hispanic	71 (28.3%)	25 (17.2%)			
	Other	15 (6%)	3 (2.1%)			
Body mass index, kg/m^2^		28.15	27.57		26.8	25.8
Blood type						
	A					
	AB					
	B					
	O					
Mechanism of death						
	Trauma	30 (12%)	28 (19.3%)		63 (48.1%)	54 (26%)
	Cerebrovascular	46 (18.3%)	17 (11.7%)		32 (24.4%)	40 (19%)
	Drug overdose	45 (17.9%)	35 (24.1%)			
	other	130 (51.8%)	65 (44.8%)		13 (9.9%)	
	anoxia				23 (17.6%)	
Diabetes mellitus		13 (5.2%)	3 (2.1%)		3 (2.4%)	
CMV serology positive						
Matching and Transplant-related characteristics						
Sex matched		191 (76.1%)	119 (82.1%)		84 (62.7%)	
Race matched		98 (39%)	76 (52.4%)			
HLA matched		30 (12.8%)	20 (15.9)			
AB0 matched		195 (77.7%)	121 (83.4%)			
CMV matched		79 (31.6%)	61 (42.4%)			
Graft cold ischemic time, min		202	208		222	

Values are presented as means. PGD, Primary Graft Dysfunction; CMV, Cytomegalovirus; HLA, Human leukocyte antigens; HM3, HeartMate 3; UNOS, United Network of Organ Sharing; ISHLT, International Society of Heart and Lung Transplantation; VA-ECMO, Venoarterial extracorporeal membrane oxygenation; LVAD, Left ventricular assist device; d-LVAD, durable LVAD; t-LVAD, temporary LVAD; LVEF, Left ventricular ejection fraction; HW, HeartWare; N/A, Not applicable.

**Table 2.** **Primary graft dysfunction after heart transplantation**.

Study characteristics		Kawabori *et al*. [14]	Thomas *et al*. [15]	Yuan *et al*. [16]
Device-related characteristics				
Device type		VA-ECMO	t-LVAD	VA-ECMO
Specific designation			CentriMag	
Patients with a circulatory support device		9	2	24
Post-support outcome and complications				
Length of circulatory support, days		10		6.9
Successful weaning from circulatory support		9 (100%)	2 (100%)	
Post-transplant hospital length of stay, days		43		
Recipient characteristics				
Recipient age, years		55.1		47.6
Sex, male		9 (100%)		18 (72%)
Body mass index, kg/m^2^		31.7		22.8
Heart failure etiology				
	Non-Ischemic	4 (44.44%)		
	Ischemic, absolute	5 (55.56%)		
	Congenital			
	Restrictive/Hypertrophic			
	Valvular			
	Other/missing/unknown			
Most recent postoperative creatinine, g/dL		1.46		1.76
Intravenous inotropes		5 (55.56%)		
Donor characteristics				
Donor age, years		35.1		
Sex, male		8 (88.89%)		
Body mass index, kg/m^2^		27.21		
Mechanism of death				
	Trauma, absolute	6 (66.66%)		
	Cerebrovascular			
	Drug overdose			
	other			
	anoxia	3 (33.33%)		
Graft LVEF <50%		0 (0%)		

CMV, Cytomegalovirus; HLA, Human leukocyte antigens; Values are presented as means; VA-ECMO, Venoarterial extracorporeal membrane oxygenation; t-LVAD, temporary left ventricular assist device; LVEF, Left ventricular ejection fraction.

### 3.1 Bridging to Heart Transplantation

#### 3.1.1 Device-Related Characteristics

We included two studies, which reported results after using a d-LVAD, one VA-ECMO study, and one t-LVAD study (Table 1). The t-LVAD study reported characteristics and results separately using the Impella 5.0 and the Impella 5.5 device [2]. Thus, we included each subgroup as a separate study in the analysis. Kilcoyne *et al*.’s registry study [11] included only patients supported with an HM3 LVAD. In contrast, the single-center study by Bedanova *et al*. [13] included patients supported with an HM2 or HW. In total, *n* = 1678 patients were included in the analysis.

#### 3.1.2 Recipient Characteristics

The d-LVAD study by Kilcoyne *et al*. [11] provided only information on device-related characteristics. The mean recipient age ranged between 44.9 years in the VA-ECMO study and 53 to 58 years in the d-LVAD and t-LVAD studies (Table 1). Both t-LVAD populations were predominantly male (80.9% and 86.9%). In the d-LVAD study by Bedanova *et al*. [13], only 45% of patients were male. The body mass index was comparable between the studies and ranged from 25.5 to 27.7 kg/m^2^. Information regarding the blood type, pretransplant mechanical ventilation, and intravenous inotropes at registration cannot be compared between studies due to a lack of reporting. Diabetes mellitus was prevalent, with 11.3% in the VA-ECMO study, 17% in the d-LVAD study by Bedanova *et al*. [13], and 31.1 to 33.8% in the t-LVAD studies.

#### 3.1.3 Donor, Matching- and Transplant-Related Characteristics

The donor age was 31 to 35 years in the t-LVAD and VA-ECMO study and 42 years in the d-LVAD study by Bedanova *et al*. [13]. The prevalence of diabetes mellitus in the donor was low, as stated, 2.4 to 5.2% in the VA-ECMO and t-LVAD studies. Neither of the d-LVAD studies states any matching characteristics. Compared to the t-LVAD studies, sex-matching was low in the VA-ECMO study, with 62.7%. The graft cold ischemic time was 202 to 208 min in the t-LVAD studies and 222 min in the VA-ECMO study.

#### 3.1.4 Outcome

**Fig. 2.**
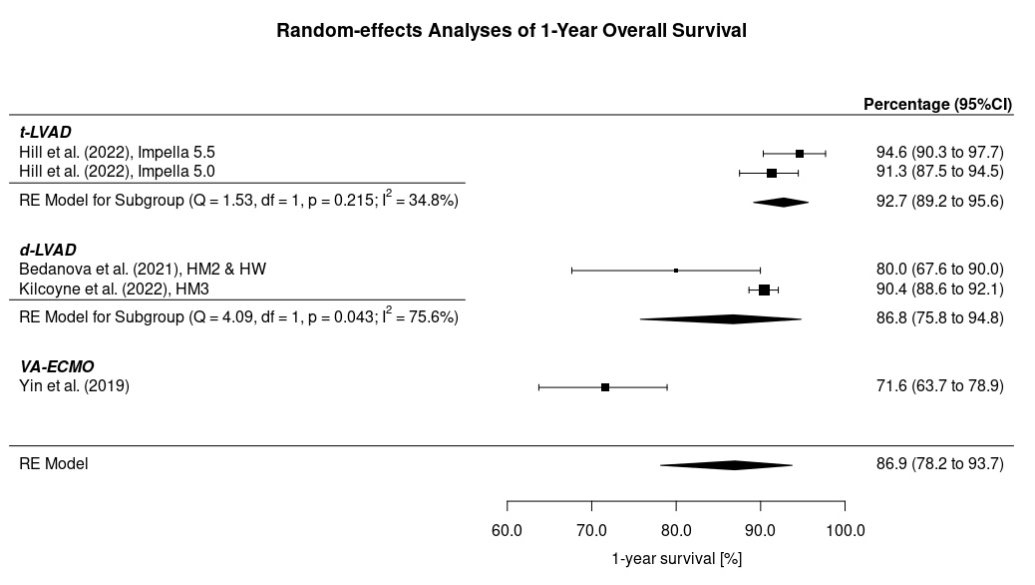
**One-year survival after heart transplantation in patients bridged with a circulatory support system**. HM2, HeartMate 2; HM3, HeartMate 3; HW, HeartWare; VA-ECMO, Venoarterial extracorporeal membrane oxygenation; LVAD, Left ventricular assist device; d-LVAD, durable LVAD; t-LVAD, temporary LVAD; LVEF, Left ventricular ejection fraction; HW, HeartWare; RE, Random effects; CI, Confidence interval.

After HTx in patients bridged to transplantation with a circulatory support system, the outcome was presented homogeneously as 1-year overall survival in all included studies (Fig. 2). The 1-year survival was 94.6% in the Impella 5.5 group and 91.3% in the Impella 5.0 group, leading to a survival of 92.7% (95% CI: 89.2 to 95.6%) after HTx in patients bridged with a t-LVAD. The 1-year survival was 80.0% and 90.4% in the d-LVAD studies, leading to a 1-year survival after HTx in patients bridged with a d-LVAD of 86.8% (95% CI: 75.8 to 94.8%). Survival after HTx in patients bridged with a VA-ECMO was 71.6% (95% CI: 63.7 to 78.9%). The 1-year survival in all included studies was 86.9% (95% CI: 78.2 to 93.7%). The statistical comparison between d-LVAD, t-LVAD, and VA-ECMO revealed that bridging with a d-LVAD (*p* = 0.027) and t-LVAD (*p* = 0.001) seems to result in a significantly higher 1-year survival probability after HTx than bridging with VA-ECMO. The 1-year survival probability of d-LVAD and t-LVAD was not significantly different (*p* = 0.210).

### 3.2 Primary Graft Dysfunction After Heart Transplantation

#### 3.2.1 Device-Related Characteristics

For treating PGD after HTx, we included three studies in total (Table 2). Two studies reported results after circulatory support with VA-ECMO, and one study reported results after circulatory support of two cases with a t-LVAD, namely CentriMag. In total, *n* = 35 patients were included.

#### 3.2.2 Recipient Characteristics

The t-LVAD study did not report information regarding donor and recipient characteristics. The recipient age ranged from 47.6 to 55.1 years in the VA-ECMO studies. Male sex ranged from 72 to 100%. The body mass index was 22.8 kg/m^2^ in the study by Yuan *et al*. [16] and 31.7 kg/m^2^ in the study by Kawabori *et al*. [14]. The most recent postoperative creatinine was 1.46 and 1.76 g/dL.

#### 3.2.3 Outcome

**Fig. 3.**
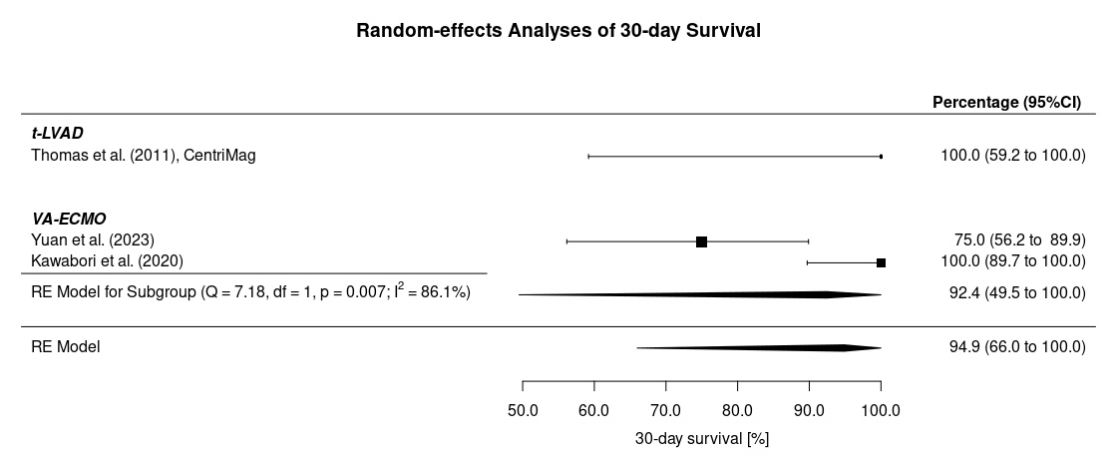
**30-day survival in patients with primary graft failure after heart transplantation treated with a circulatory support system**. VA-ECMO, Venoarterial extracorporeal membrane oxygenation; t-LVAD, temporary left ventricular assist device; RE, Random effects; CI, Confidence interval.

The outcome after HTx in patients with PGD treated with a circulatory support system was presented homogeneously as 30-day survival in all included studies (Fig. 3). Based on the very limited data, the 30-day survival rate in the study by Yuan *et al*. [16] was 72.5%, equivalent to 17.4 of 24 patients. Based on the survival curve, which was also provided in the publication, we assumed a 75% 30-day survival, which would correspond to 18 of 24 patients. The 30-day survival rate in patients who were treated with t-LVAD was 100% (95% CI: 59.2 to 100%). In patients supported with VA-ECMO for PGD, the outcome was 75% and 100%, leading to a 30-day survival of 92.4% (95% CI: 66 to 100%).

## 4. Discussion

For bridging to HTx, t-LVAD support seems to lead to at least equivalent 1-year survival rates after HTx as d-LVAD support. This might be because the implanted t-LVAD devices provide sufficient circulatory support comparable to a d-LVAD. Thus, both support types seem to stabilize the transplant candidate equally during bridging. However, the survival rate in patients bridged with VA-ECMO appears to be lower, although they were almost ten years younger than those in the other studies. A worse outcome in patients bridged with VA-ECMO compared to d-LVAD bridging is consistent with a published article by Lund *et al*. [4]. Nevertheless, a comparison with a t-LVAD has been missing so far because, at the time of the publication of the mentioned article, bridging with a t-LVAD had not been performed. This gap in knowledge has now been closed. ECMO therapy increases the risk of neurologic complications, bleeding, thrombotic events, renal failure, and vascular-access complications [17, 18, 19, 20, 21]. As a result, the status of transplant candidates bridged with VA-ECMO might be worse due to possibly decreased kidney and liver function or coagulation problems compared to patients who were bridged with a d-LVAD. This could possibly also affect the post-transplant outcome. Besides possible outcome differences between the circulatory support systems, the survival after HTx in general appears relatively high. A high survival after HTx can be based on an intended analysis of patients with a good outcome, young or carefully selected recipients, or improvements in donor or recipient management. This first aspect is unlikely to have appeared because the registry studies, which contributed to the high number of patients, described a well-elaborated inclusion protocol. The mean recipient age, when reported, was also not surprisingly low.

Nevertheless, the recipient age was unknown in the d-LVAD study by Kilcoyne *et al*. [11], which included over a thousand patients. Donor and recipient management are important for survival. However, the time frame of the included studies is very comparable, so no device-type-specific bias should have occurred.

The d-LVAD study by Bedanova *et al*. [13] showed a worse 1-year survival compared to the registry d-LVAD study and compared to the t-LVAD studies. However, it also needs to be mentioned that in the study by Bedanova *et al*. [13], the donors were about ten years older than the recipients bridged with a t-LVAD. Considering that donor age is a risk factor for 1-year post-transplant mortality, the higher donor age could explain the differing outcomes of the studies [4]. The t-LVAD study predominantly consists of male recipients, who might be less likely to develop graft failure than female recipients based on the literature [22, 23]. However, broad evidence that female sex is a risk factor for impaired early survival is missing. The impact of recipient diabetes mellitus, which was more prevalent in the t-LVAD study, on post-transplant survival has been investigated multiple times. However, based on the literature, conclusions remain unclear [24, 25]. It is also important to highlight that the d-LVAD study by Bedanova *et al*. [13] is single-center, while the t-LVAD studies are registry studies. Registry studies allow the inclusion of a large amount of data collected from multiple centers. However, the data quality depends on the individual center and thus might be heterogeneous [26]. In the case of a single-center study, data quality tends to be homogenous. At the same time, the therapy quality is based on the individual experience of a single center, and most likely, patient selection is restricted.

It would be interesting to reanalyze outcomes after HTx in patients bridged with a t-LVAD, which is intended and approved for longer use and thus more appropriate as a bridging device when available on the market. Finally, the impact of the support device on the high-urgency listing status of a transplant candidate might also have an impact, as only d-LVAD implantation leads to losing the high-urgency status.

Only a few studies exist on treating post-transplant PGD with a circulatory support system. Additionally, the number of patients is low, consisting of only two cases with a t-LVAD. Consequently, the interpretability of results regarding this part of the meta-analysis is reduced. Furthermore, data representing the outcome of patients after PGD treated with an Impella 5.0 or 5.5 system, which is one of the most frequently used t-LVADs nowadays, has not been published yet. Furthermore, two of three studies had almost no donor- and transplant-related information. Thus, donor- or recipient-associated factors that might interfere with the circulatory support system cannot be identified. The most recent postoperative creatinine was higher in the VA-ECMO study by Yuan *et al*. [16], which could partially explain the worse outcome [4]. The majority of included studies in both analyses are of a single-arm observational nature and can underlie a certain level of bias. Some of the included studies explicitly excluded multiorgan transplants, which leads to a stabilization of results. Nevertheless, in future studies, when larger data sets are available, inclusion of studies should be restricted to those that excluded multiorgan transplantation to exclude any possibility of bias.

## 5. Conclusion

D-LVAD, t-LVAD, and VA-ECMO are used to bridge patients to HTx. VA-ECMO and t-LVADs were used in the included studies to treat PGD post-HTx. The type of circulatory support device impacts the 1-year survival after HTx. Both d-LVAD and t-LVAD seem to result in a higher 1-year survival rate after HTx than VA-ECMO. However, d-LVAD and t-LVAD appear to have a comparable 1-year survival probability. Circulatory support for bridging to HTx is much more investigated, especially by registry studies, than for PGD after HTx. In PGD, VA-ECMO seems to be predominantly used. Unfortunately, the device-specific outcomes after the treatment of PGD cannot be compared due to low patient numbers. However, more prospective clinical studies investigating circulatory support devices in PGD after HTx need to be performed.

## Data Availability

Data is already included in this publication.

## Abbreviations

HTx, heart transplantation; d-LVAD, durable left ventricular assist device; t-LVAD, temporary left ventricular assist devices; VA-ECMO, veno-arterial extracorporeal membrane oxygenation; PGD, primary graft dysfunction; PRISMA, Preferred Reporting Items for Systematic Reviews and Meta-analysis statement.

## Supplementary Material

Supplementary material associated with this article can be found, in the online version, at https://doi.org/10.31083/RCM45064.
