# Supplementary material for: Meta-Analysis of Durable Compared to Temporary Left Ventricular Assist Devices Compared to Venoarterial Extracorporeal Membrane Oxygenation for Bridging to Heart Transplantation or Treatment of Primary Graft Dysfunction
Source: Rev Cardiovasc Med. 2025 Dec 16;26(12):45064. doi: 10.31083/RCM45064 (PMC12780990; doi:10.31083/RCM45064)
Supplement: Supplementary file 1 [file 2153-8174-26-12-45064-s1.zip › Supplementary Material.docx]

*Search strategy*

The following search strategy was predefined for the bridging to HTx meta-analysis: ((heart transplantation) AND ((bridge) OR (bridging) OR (bridge to transplant) OR (bridging to transplant)) AND ((ECMO) OR (extracorporeal membrane oxygenation) OR (left ventricular assist device) OR (LVAD) OR (heart mate) OR (heart ware) OR (impella)) AND ((controlled trial) OR (controlled study) OR (registry study))). The following search strategy was predefined for the PGD after HTx meta-analysis: ((heart transplantation) AND ((primary graft failure) OR (primary graft dysfunction) OR (PGD) OR (PGF)) AND ((ECMO) OR (extracorporeal membrane oxygenation) OR (left ventricular assist device) OR (LVAD) OR (heart mate) OR (heart ware) OR (impella)) AND ((controlled trial) OR (controlled study) OR (registry study))).
