# Supplementary material for: Meta-Analysis of Durable Compared to Temporary Left Ventricular Assist Devices Compared to Venoarterial Extracorporeal Membrane Oxygenation for Bridging to Heart Transplantation or Treatment of Primary Graft Dysfunction
Source: Rev Cardiovasc Med. 2025 Dec 16;26(12):45064. doi: 10.31083/RCM45064 (PMC12780990; doi:10.31083/RCM45064)
Supplement: Supplementary file 1 [file 2153-8174-26-12-45064-s1.zip › Supplementary Table 1.docx]

**Supplementary Table 1. National Institutes of Health Quality Assessment Tool for Observational Cohort and Cross-Sectional Studies** [1].

| **[2]** | **[3]** | **[4]** | **[5]** | **[6]** | **[7]** | **[8]** |
| --- | --- | --- | --- | --- | --- | --- |
| **1. Was the research question or objective in this paper clearly stated?** | | | | | | |
| Yes | Yes | Yes | Yes | Yes | Yes | Yes |
| **2. Was the study population clearly specified and defined** | | | | | | |
| Yes | Yes | Yes | Yes | Yes | Yes | Yes |
| **3. Was the participation rate of eligible persons at least 50%?** | | | | | | |
| N/A | N/A | N/A | N/A | N/A | N/A | N/A |
| **4. Were all the subjects selected or recruited from the same or similar populations (including the same time period)? Were the inclusion and exclusion criteria for being in the study prespecified and applied uniformly to all participants?** | | | | | | |
| Yes | Yes | Yes | Yes | Yes | Yes | Yes |
| **5. Was a sample size justification, power description, or variance and effect estimates provided?** | | | | | | |
| N/A | N/A | N/A | N/A | N/A | N/A | N/A |
| **6. For the analyses in this paper, were the exposure(s) of interest measured prior to the outcome(s) being measured?** | | | | | | |
| N/A | N/A | N/A | N/A | N/A | N/A | N/A |
| **7. Was the timeframe sufficient so that one could reasonably expect to see an association between exposure and outcome if it existed?** | | | | | | |
| Yes | Yes | Yes | Yes | Yes | Yes | Yes |
| **8. For exposures that can vary in amount or level, did the study examine different levels of the exposure as related to the outcome (e.g., categories of exposure, or exposure measured as a continuous variable)?** | | | | | | |
| N/A | N/A | N/A | N/A | N/A | N/A | N/A |
| **9. Were the exposure measures (independent variables) clearly defined, valid, reliable, and implemented consistently across all study participants?** | | | | | | |
| Yes | Yes | Yes | Yes | Yes | Yes | Yes |
| **10. Was the exposure(s) assessed more than once over time?** | | | | | | |
| N/A | N/A | N/A | N/A | N/A | N/A | N/A |
| **11. Were the outcome measures (dependent variables) clearly defined, valid, reliable, and implemented consistently across all study participants?** | | | | | | |
| Yes | Yes | Yes | Yes | Yes | Yes | Yes |
| **12. Were the outcome assessors blinded to the exposure status of participants?** | | | | | | |
| NS | NS | NS | NS | NS | NS | NS |
| **13. Was the loss to follow-up after baseline 20% or less?** | | | | | | |
| N/A | N/A | N/A | N/A | N/A | N/A | N/A |
| **14. Were key potential confounding variables measured and adjusted statistically for their impact on the relationship between exposure(s) and outcome(s)?** | | | | | | |
| N/A | N/A | N/A | N/A | N/A | N/A | N/A |
| **Quality (good/fair/poor) Reviewer 1** | | | | | | |
| Good | Good | Good | Good | Good | Good | Good |
| **Quality (good/fair/poor) Reviewer 2** | | | | | | |
| Good | Good | Good | Good | Good | Good | Good |

References

[1] National Institutes of Health. Study Quality Assessment Tools. [March 3rd 2024]; Available from: https://www.nhlbi.nih.gov/health-topics/study-quality-assessment-tools.

[2] Hill MA, Kwon JH, Shorbaji K, Kilic A. Waitlist and transplant outcomes for patients bridged to heart transplantation with Impella 5.0 and 5.5 devices. Journal of cardiac surgery 2022;37(12):5081–9.

[3] Kilcoyne MF, Huckaby LV, Hashmi Z, Witer L, Pope N, Houston BA et al. The HeartMate 3 left ventricular assist device as a strategy to bridge to transplant. Journal of cardiac surgery 2022;37(12):4713–8.

[4] Yin MY, Wever-Pinzon O, Mehra MR, Selzman CH, Toll AE, Cherikh WS et al. Post-transplant outcome in patients bridged to transplant with temporary mechanical circulatory support devices. The Journal of heart and lung transplantation the official publication of the International Society for Heart Transplantation 2019;38(8):858–69.

[5] Bedanova H, Pazdernik M, Sonka M, Zhi C, Krejci J, Novakova M et al. Effects of implanting a long-term left ventricle assist device on post-transplant outcomes. The International journal of artificial organs 2021;44(1):39–45.

[6] Kawabori M, Mastroianni MA, Zhan Y, Chen FY, Rastegar H, Warner KG et al. A case series: the outcomes, support duration, and graft function recovery after VA-ECMO use in primary graft dysfunction after heart transplantation. Journal of artificial organs the official journal of the Japanese Society for Artificial Organs 2020;23(2):140–6.

[7] Thomas HL, Dronavalli VB, Parameshwar J, Bonser RS, Banner NR. Incidence and outcome of Levitronix CentriMag support as rescue therapy for early cardiac allograft failure: a United Kingdom national study. European journal of cardio-thoracic surgery official journal of the European Association for Cardio-thoracic Surgery 2011;40(6):1348–54.

[8] Yuan L, Ma W, Cui J, Liu J, Yang Z, Yang S et al. Mildly Elevated Pulmonary Artery Systolic Pressure is Associated with Extracorporeal Membrane Oxygenation Support after Heart Transplantation. Journal of cardiac surgery 2023;2023:1–7.
